# How young radiologists use contrast media and manage adverse reactions: an international survey

**DOI:** 10.1186/s13244-024-01658-z

**Published:** 2024-03-26

**Authors:** Domenico Albano, Carmen Mallardi, Saif Afat, Paulo Moraes Agnollitto, Damiano Caruso, Roberto Cannella, Serena Carriero, Kalina Chupetlovska, Paola Clauser, Tommaso D’Angelo, Domenico De Santis, Marco Dioguardi Burgio, Ivo Dumic-Cule, Salvatore Claudio Fanni, Stefano Fusco, Marco Gatti, Salvatore Gitto, Sonja Jankovic, Tsvetomir Karagechev, Michail E. Klontzas, Emmanouil Koltsakis, Doris Leithner, Vid Matišić, Giuseppe Muscogiuri, Ralitsa Penkova, Michela Polici, Francesca Serpi, Carmelo Sofia, Ziga Snoj, Tugba Akinci D’Antonoli, Federica Vernuccio, João Vieira, Ana Catarina Vieira, Mirjam Wielema, Marta Zerunian, Carmelo Messina

**Affiliations:** 1 IRCCS Istituto Ortopedico Galeazzi, Milan, Italy; 2 Dipartimento Di Scienze Biomediche, Chirurgiche Ed Odontoiatriche, Università Degli Studi Di Milano, Milan, Italy; 3 Scuola Di Specializzazione in Radiodiagnostica, Università Degli Studi Di Milano, Milan, Italy; 4 Department of Diagnostic and Interventional Radiology, Eberhard Karls University Tuebingen, Tuebingen, Germany; 5 Ribeirão Preto Medical School, Radiology Division of the Department of Medical Imaging, Hematology and Clinical Oncology, University of São Paulo, São Paulo, Ribeirão Preto Brazil; 6 Department of Medical Surgical Sciences and Translational Medicine, Sant’Andrea University Hospital, Sapienza - University of Rome, Rome, Italy; 7 Section of Radiology, Department of Biomedicine, Neuroscience and Advanced Diagnostics (BiND), University of Palermo, Palermo, Italy; 8 Department of Radiology and Interventional Radiology, Foundation IRCCS Ca’ Granda Ospedale Maggiore Policlinico, Milan, Italy; 9 Department of Radiology, The Netherlands Cancer Institute, Amsterdam, The Netherlands; 10 Department of Biomedical Imaging and Image-Guided Therapy, Division of General and Pediatric Radiology, Medical University of Vienna, Vienna, Austria; 11 Diagnostic and Inverventional Radiology Unit, Department of Biomedical Sciences and Morphological and Functional Imaging, University of Messina, Messina, Italy; 12 Department of Radiology and Nuclear Medicine, Erasmus MC, Rotterdam, The Netherlands; 13 Department of Radiology, Hôpital Beaujon, AP-HP.Nord, 100 Boulevard du Général Leclerc, 92110 Clichy, France; 14 Université Paris Cité, INSERM, Centre de Recherche Sur L’inflammation, 75018 Paris, France; 15 Department of Diagnostic and Interventional Radiology, University Hospital Centre Zagreb, Kispaticeva 12, 10000 Zagreb, Croatia; 16 University North, 104 Brigade 3, 42000 Varazdin, Croatia; 17 Department of Translational Research, Academic Radiology, University of Pisa, Pisa, Italy; 18 Department of Biomedical Sciences for Health, Università Degli Studi Di Milano, Milan, Italy; 19 Radiology Unit, Department of Surgical Sciences, University of Turin, Turin, Italy; 20 Center for Radiology, University Clinical Center Nis, Nis, Republic of Serbia; 21 Department of Radiology, Acibadem City Clinic Tokuda Hospital, Sofia, Bulgaria; 22 Department of Radiology, School of Medicine, University of Crete, Heraklion, Crete, Greece; 23 Department of Medical Imaging, University Hospital of Heraklion, Heraklion, Crete, Greece; 24 Department of Radiology, Karolinska University Hospital of Stockholm, Stockholm, Sweden; 25 Department of Radiology, NYU Grossman School of Medicine, New York, NY USA; 26 St. Catherine Specialty Hospital, 10000 Zagreb, Croatia; 27 Department of Radiology, ASST Papa Giovanni XXIII, Bergamo, Italy; 28 Radiology Department, Acibadem City Clinic Tokuda Hospital, 51B Nikola Y. Vaptsarov Blvd, Sofia, 1407 Bulgaria; 29 PhD School in Traslational Medicine and Oncology, Department of Medical Surgical Sciences and Translational Medicine, Faculty of Medicine and Psychology, “Sapienza” University of Rome, Rome, Italy; 30 Radiology Institute, University Medical Centre Ljubljana, Zaloška 7, Ljubljana, Slovenia; 31 Institute of Radiology and Nuclear Medicine, Cantonal Hospital Baselland, Liestal, Switzerland; 32 Radiology, Hospital Divino Espírito Santo, Ponta Delgada, Portugal; 33 Radiology Department, Hospital CUF Porto, Porto, Portugal; 34 Faculty of Medicine, University of Porto, Porto, Portugal; 35 Department of Radiology, Canisius Wilhelmina Hospital, Nijmegen, The Netherlands

**Keywords:** Adverse drug reaction, Contrast medium, Education, Safety, Training

## Abstract

**Objectives:**

To collect real-world data about the knowledge and self-perception of young radiologists concerning the use of contrast media (CM) and the management of adverse drug reactions (ADR).

**Methods:**

A survey (29 questions) was distributed to residents and board-certified radiologists younger than 40 years to investigate the current international situation in young radiology community regarding CM and ADRs. Descriptive statistics analysis was performed.

**Results:**

Out of 454 respondents from 48 countries (mean age: 31.7 ± 4 years, range 25–39), 271 (59.7%) were radiology residents and 183 (40.3%) were board-certified radiologists. The majority (349, 76.5%) felt they were adequately informed regarding the use of CM. However, only 141 (31.1%) received specific training on the use of CM and 82 (18.1%) about management ADR during their residency. Although 266 (58.6%) knew safety protocols for handling ADR, 69.6% (316) lacked confidence in their ability to manage CM-induced ADRs and 95.8% (435) expressed a desire to enhance their understanding of CM use and handling of CM-induced ADRs. Nearly 300 respondents (297; 65.4%) were aware of the benefits of contrast-enhanced ultrasound, but 249 (54.8%) of participants did not perform it. The preferred CM injection strategy in CT parenchymal examination and CT angiography examination was based on patient’s lean body weight in 318 (70.0%) and 160 (35.2%), a predeterminate fixed amount in 79 (17.4%) and 116 (25.6%), iodine delivery rate in 26 (5.7%) and 122 (26.9%), and scan time in 31 (6.8%) and 56 (12.3%), respectively.

**Conclusion:**

Training in CM use and management ADR should be implemented in the training of radiology residents.

**Critical relevance statement:**

We highlight the need for improvement in the education of young radiologists regarding contrast media; more attention from residency programs and scientific societies should be focused on training about contrast media use and the management of adverse drug reactions.

**Key points:**

• This survey investigated training of young radiologists about use of contrast media and management adverse reactions.

• Most young radiologists claimed they did not receive dedicated training.

• An extreme heterogeneity of responses was observed about contrast media indications/contraindications and injection strategy.

**Graphical Abstract:**

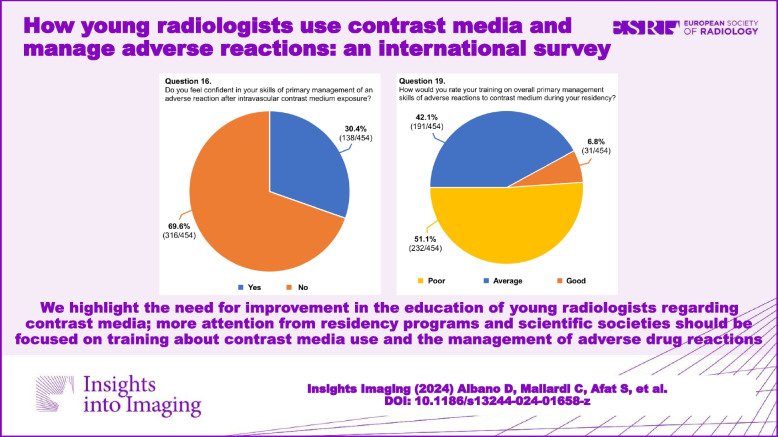

## Introduction

Advancements in technology and knowledge have led radiologists to an ever-increasing demand for accurate and comprehensive imaging diagnosis [1]. Young radiologists in particular find themselves at the forefront of this transformative era. Their role in healthcare is more critical than ever, as they are entrusted with the task of interpreting medical images of outpatient, inpatient, and emergency examinations to guide clinical decisions. Central to this practice is the use of contrast media (CM) that enhances the visibility of specific anatomical structures and pathological conditions during imaging procedures. The pivotal role of CM is proven by the substantial growth in using contrast enhanced imaging methods, with half of CT and MRI examinations including its use, mostly iodinated and gadolinium-based CM [2]. Although CMs are generally considered safe, adverse drug reactions (ADR) still occur, presenting in up to 1% of examinations [3]. These include severe acute CM reactions that need urgent treatment. ADRs may present with a drop in blood pressure, dyspnea, loss of consciousness, and/or cardiac arrest [2, 4]. Iodinated CM is the third most frequent cause of ADR, after non-steroidal anti-inflammatory and chemotherapeutic drugs [2, 5]. In addition to ADR, a non-negligible grade of toxicity of CM must be considered, including the well-known nephrotoxicity of iodinated CM and nephrogenic systemic fibrosis induced by gadolinium-based CM [6]. These adverse effects potentially represent life-threatening conditions and together with the deposition of these molecules in the human tissues, have clinical effects that remain not entirely clear [7, 8]. As a matter of fact, the knowledge of indications and contraindications, the choice of volume and type of CM, the need for premedication, and the management of ADR and contrast extravasation are basic skills radiologists should have and improve over time in their daily clinical practice.

Radiology training is mostly focused on learning normal and pathological imaging findings of various diseases. However, young radiologists are called to be proficient in the utilization of CM; therefore, their specific education on this topic along with challenges, opportunities, and responsibilities in harnessing the full potential of these substances cannot be overstated. Especially in the emergency setting, the awareness of the correct use of CM and the management of ADR become essential.

This paper reports real-world data collected from an international survey focused on the knowledge and self-perception of young radiologists concerning the use of CM, as well as awareness and management of ADR. The aim of this article was to obtain insight into the training of young radiologists on the use of CM.

## Methods

### Study design

Institutional Review Board approval was not needed for this survey study, as no patient was involved. The authors prepared an online survey for widespread distribution. The questionnaire was sent via email to board-certified radiologists and radiology residents younger than 40 years old across European and non-European Countries on September 4, 2023. The questionnaire was further distributed using social media channels of the different European radiology societies. The online survey was available till October 6. Similar to previous online surveys [9–13], we used the free online tool “Microsoft Forms” to create the survey and to collect all data, consisting of 29 questions, of which five were open-ended questions, while 24 were closed-ended questions, either with unique answers or multiple-choice selections. The questionnaire was developed by the participating panelists using a consensus process, where new questions were proposed and agreed in consensus by 35 panel members from 16 countries. Demographic data and information on training institutions were collected. The survey itself was focused on the knowledge, self-perception, and training of young radiologists and trainees on the use of CM and the management of ADRs. Data were collected and managed in aggregated form to ensure anonymity. The full list of questions and answers is reported in Table 1.

**Table 1:** Full list of questions and answers (total participants = 454)

Question	Answer
1. How old are you?	31.7 ± 4 years (range 25–39)
2. In which country did you receive (or do you receive) your radiology training?	See Table 2
3. What is your country of origin?	See Table 2
4. Are you a resident or a certified radiologist?	Residents: 271/454 (59.7%)
	Radiologists: 183/454 (40.3%)
5. What type of hospital do you work in?	University hospital: 321/454 (70.7%)
	Larger community hospital: 84/454 (18.5%)
	Small community hospital: 22/454 (4.8%)
	Private hospital: 15/454 (3.3%)
	Private practice: 12/454 (2.6%)
6. What percentage of CT/MR are performed with CM at your Institution in a year?	> 50%: 336/454 (74.0%)
	< 50%: 118/454 (26.0%)
7. How many different CM (molecules) do you usually use for CT in your practical activity?	0: 2/454 (0.4%)
	1: 71/454 (15.6%)
	2: 206/454 (45.4%)
	3: 100/454 (22.0%)
	> 4: 75/454 (16.5%)
8. How many different CM (molecules) do you usually use for MR in your practical activity?	0: 8/454 (1.8%)
	1: 58/454 (12.8%)
	2: 196/454 (43.2%)
	3: 117/454 (25.8%)
	> 4: 75/454 (16.5%)
9. Are you well informed about the appropriate use of CM in diagnostic/interventional procedures?	Yes: 349/454 (76.5%)
	No: 107/454 (23.5%)
10. How many severe adverse reactions (e.g., pulmonary edema, respiratory arrest, convulsions, cardiac arrest, cardiogenic shock) did you witness during your working practice in the past 12 months?	None: 234/454 (51.5%)
	Less than 5: 213/454 (46.9%)
	6 to 20: 7/454 (1.5%)
11. Do you know the risk factors associated with increased probability of developing an adverse reaction after administration of CM?	Yes: 379/454 (83.5%)
	No: 75/454 (16.5%)
12. Are you familiar with the safety protocols for managing adverse reactions to CM established by your national or international Societies of Radiology, Urology or Anesthesia?	Yes: 266/454 (58.6%)
	No: 188/454 (41.4%)
13. Do you know when premedication should be used in patients with allergies?	Yes: 386/454 (85.0%)
	No: 68/454 (15.0%)
14. Did you have patients, coming for diagnostic or interventional procedures with CM, who have had non-severe allergic reactions to drugs other than CM (e.g., antibiotics)?	Yes: 311/454 (68.5%)
	No: 143/454 (31.5%)
15. In case of history of severe adverse reaction to CM, do you have to choose an alternative diagnostic procedure?	Always: 194/454 (42.7%)
	I don't know: 31/454 (6.8%)
	Never: 11/454 (2.4%)
	Only in certain cases: 218/454 (48.0%)
16. Do you feel confident in your skills of primary management of an adverse reaction after intravascular CM exposure?	Yes: 138/454 (30.4%)
	No: 316/454 (69.6%)
17. Is intravenous CM injection contraindicated during breast feeding?	I am not sure: 79/454 (17.4%)
	No: 288/454 (63.4%)
	Only in exceptional circumstances: 56/454 (12.3%)
	Yes: 31/454 (6.8%)
18. Is intravenous CM injection contraindicated during pregnancy?	I am not sure: 97/454 (21.4%)
	No: 155/454 (34.1%)
	Only in exceptional circumstances: 88/454 (19.4%)
	Yes: 114 (25.1%)
19. How would you rate your training on overall primary management skills of adverse reactions to CM during your residency?	Average: 191/454 (42.1%)
	Good: 31/454 (6.8%)
	Poor: 232/454 (51.1%)
20. Would you like to improve your knowledge about the use of CM and the management of adverse reactions?	Yes: 435/454 (95.8%)
	No: 19/454 (4.2%)
21. Did you have a dedicated training for CM agents use during your residency?	Yes: 141/454 (31.1%)
	No: 313/454 (68.9%)
22. Did you have a dedicated training for management of severe adverse reactions to CM during your residency?	Yes: 82/454 (18.1%)
	No: 372/454 (81.9%)
23. Are you facing/aware of new technological development in CM field?	Yes: 142/454 (31.3%)
	No: 312 (68.7%)
24. In your institution, who decides the amount and the type of CM (molecule) to be injected for each patient?	Chief of radiology department: 4/454 (0.9%)
	Radiologist: 282/454 (62.1%)
	Nurse: 8/454 (1.8%)
	Radiology Resident: 59/454 (13.0%)
	Technician: 101/454 (22.2%)
25. In CT angiography examinations, which of the following CM injection strategy do you use?	A predetermined fixed amount: 116/454 (25.6%)
	Based on iodine delivery rate (IDR): 122/454 (26.9%)
	Based on patient’s lean body weight: 160/454 (35.2%)
	Based on scan time: 56/454 (12.3%)
26. In CT parenchymal examinations, which of the following CM injection strategy do you use?	A predetermined fixed amount: 79/454 (17.4%)
	Based on iodine delivery rate (IDR): 26/454 (5.7%)
	Based on patient's lean body weight: 318/454 (70.0%)
	Based on scan time: 31/454 (6.8%)
27. How is calculated the amount of CM to be injected in CT examinations?	Simple calculator: 164/454 (36.1%)
	Pre-fixed tables: 98 (21.6%)
	Predetermined fixed amount: 87/454 (19.2%)
	Smartphone apps: 93/454 (20.5%)
	Approximated based on patient's weight: 4/454 (0.9%)
	I don’t know: 3/454 (0.7%)
	Calculator at CT: 5/454 (1.1%)
28. Are you aware of the advantages of the use of CM in ultrasound (CEUS) in specific settings when compared to contrast-enhanced CT/MRI?	I am not sure: 71/454 (15.6%)
	No: 86/454 (18.9%)
	Yes: 297/454 (65.4%)
29. Do you perform contrast-enhanced ultrasound (CEUS) at your Institution?	Yes: 205 (45.2%)
	No: 249 (54.8%)

### Data analysis

The dataset was analyzed by two board-certified radiologists (D.A. and C.M.) with experience in survey studies. Descriptive statistics were used, and results reported as means ± standard deviation and percentages. A sub-analysis was done to compare the replies from residents and board-certified radiologists using a *χ*^2^ test. The SPSS (IBM Corp. Released 2020. IBM SPSS Statistics for Windows, Version 27.0. Armonk, NY: IBM Corp) was used for statistical analysis. A further sub-analysis was performed comparing the replies from participants of the five most represented countries. A *p*-value less than 0.05 was considered as statistically significant.

## Results

**Fig. 1:**
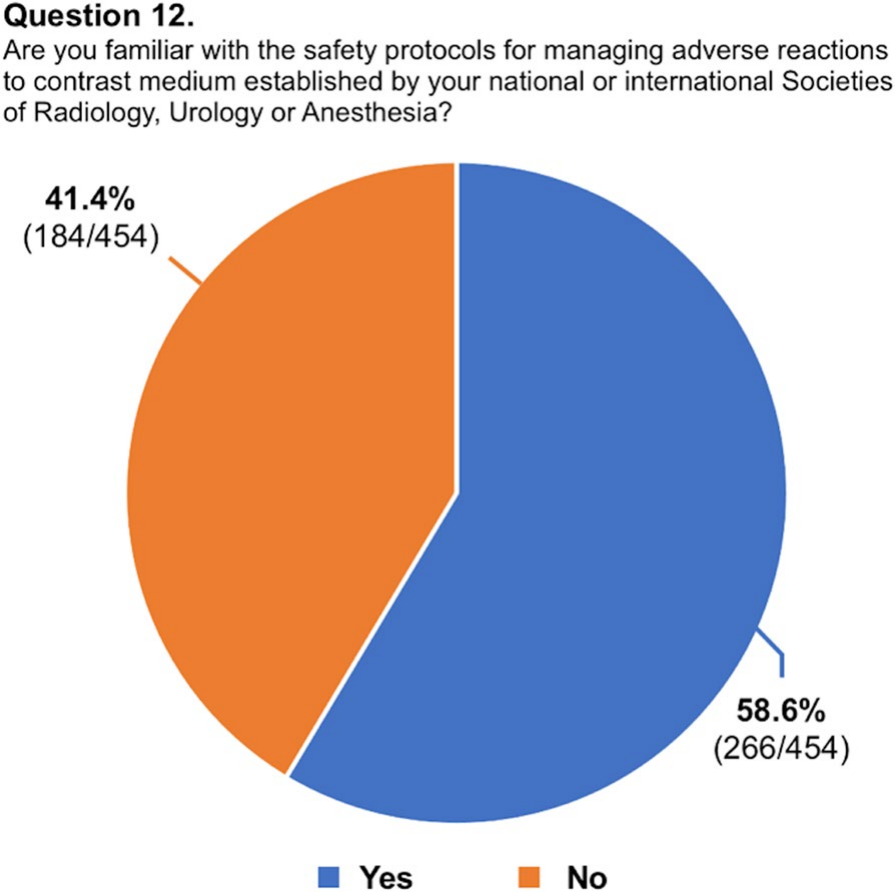
Graphical distribution of replies to question 12

**Fig. 2:**
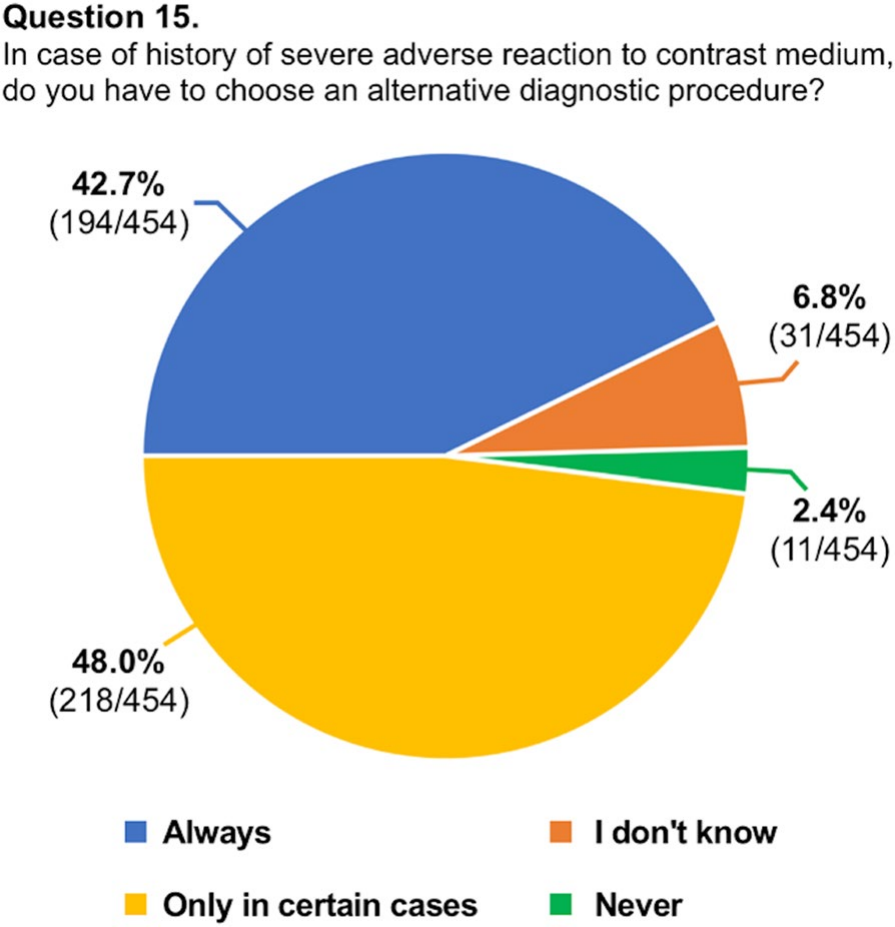
Graphical distribution of replies to question 15

**Fig. 3:**
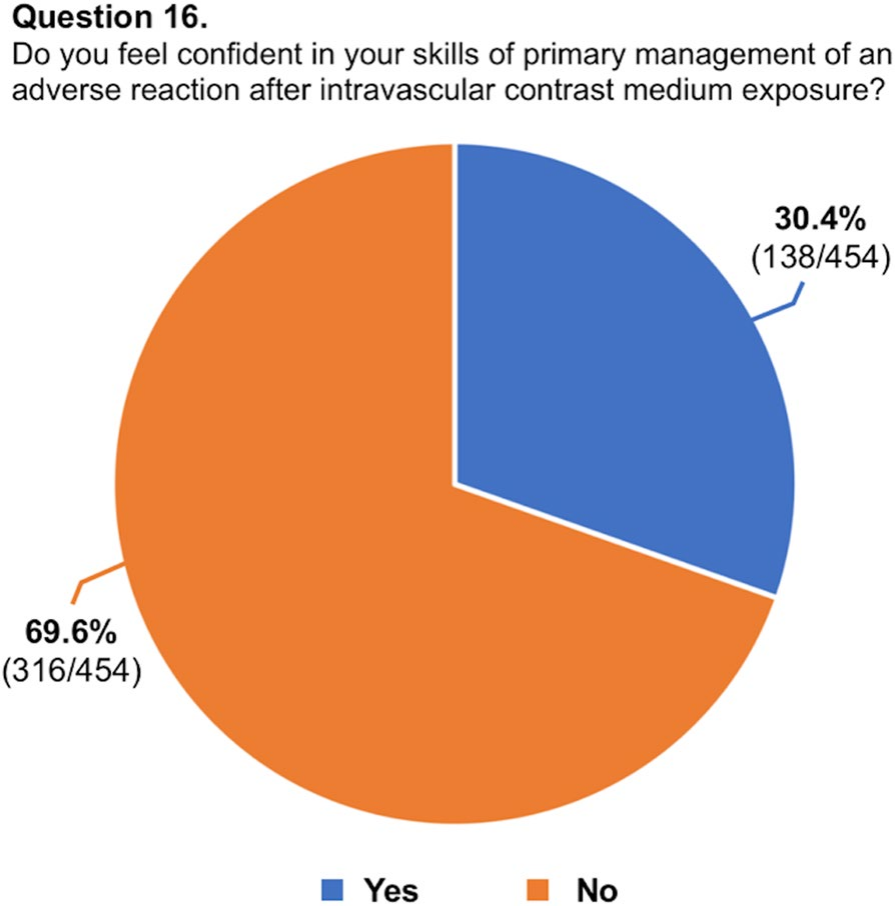
Graphical distribution of replies to question 16

**Fig. 4:**
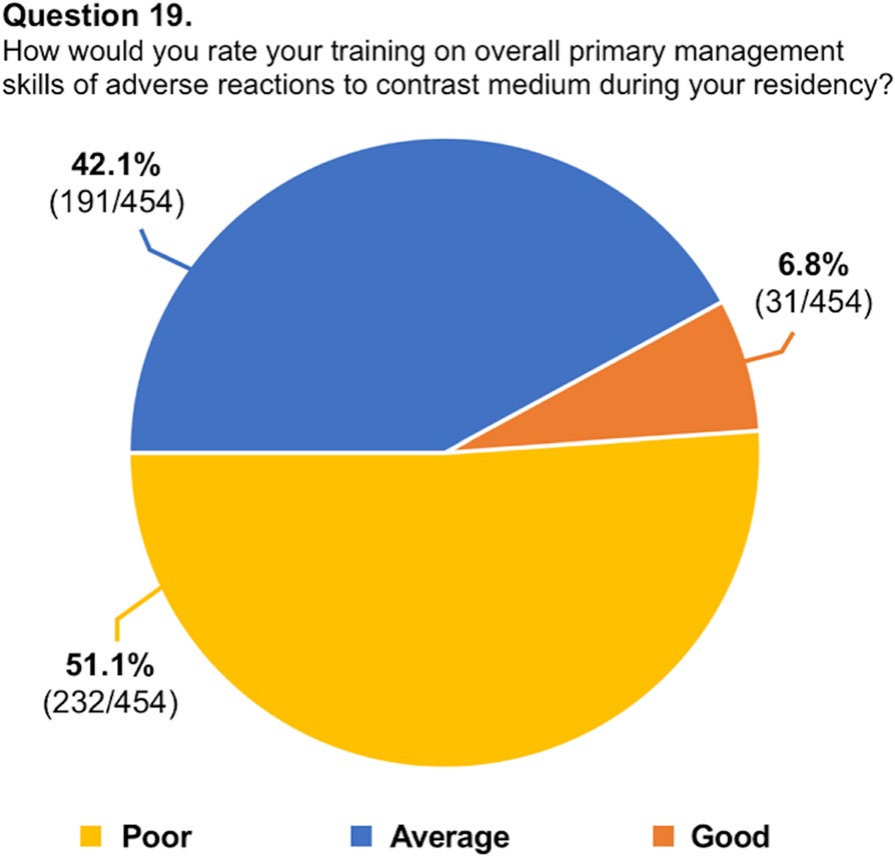
Graphical distribution of replies to question 19

A total of 454 respondents (mean age: 31.7 ± 4 years, range 25–39) from 48 countries (38 countries of radiology training institutions) replied to the questionnaire and were included in our analysis. One third of the respondents studied or practiced in Italy, while for the remaining countries, the number of respondents was better distributed. The list of countries of origin and residency is fully reported in Table 2. Most of the respondents were radiology residents (*n* = 271, 59.7%), while 183 respondents were board-certified radiologists (40.3%). Most participants (70.7%) worked in university hospitals, while 18.5% worked in large community hospitals. Participants reported that in their institutions, two-thirds of CT/MR scans are performed with CM. In approximately half of the institutions, both CT and MR were performed using two different CM molecules. Most respondents (76.5%) reported that they were adequately informed regarding the use of CM in diagnostic and interventional procedures, 83.5% were aware of the risk factors associated with ADR to CM, and 58.6% were familiar with the safety protocols stated by international scientific societies. Approximately half of respondents witnessed less than five severe ADR to CM in the past 12 months. In patients with a previous severe ADR to CM presenting with a new request of contrast-enhanced examination, respondents were split into 42.7% who always choose an alternative diagnostic procedure and 48.0% who do that just in certain cases. Only 30.4% of participants felt confident in managing an ADR to CM, 31.1% received specific training on the use of CM during their residency, and only 18.1% about the management of ADR, with 93.2% rating their training on primary management of ADR during residency as poor-to-average. A striking majority of 95.6% expressed a desire to enhance their understanding of CM use and the handling of its adverse effects. While the majority (63.4%) of respondents acknowledged that intravenous CM injection is not contraindicated during breastfeeding, 34.1% and 25.1% assumed that it is not contraindicated or is contraindicated in pregnancy, respectively. The remaining 40.8% were not sure or stated that just in exceptional circumstances, it would be contraindicated. The preferred CM injection strategy in CT parenchymal examination and CT angiography was based on the patient’s lean body weight at 70% and 35.2%, a predeterminate fixed amount at 17.4% and 25.6%, iodine delivery rate at 5.7% and 26.9%, and scan time in 6.8% and 12.3%, respectively. Finally, 65.4% were aware of the benefits of contrast-enhanced ultrasound, but 54.8% of participants did not perform it in their institution. Questions #12, #15, #16, and #19 were reported in graphics (Figs. 1, 2, 3 and 4).

**Table 2:** Full list of countries of origin and radiology training institutions of respondents

Country	No. of country of residency (%)	No. of country of origin (%)
Italy	161 (35.5%)	160 (35.2%)
Slovenia	45 (9.9%)	43 (9.5%)
Croatia	29 (6.4%)	29 (6.4%)
Serbia	24 (5.3%)	25 (5.5%)
Spain	21 (4.6%)	19 (4.2%)
Bulgaria	20 (4.4%)	19 (4.2%)
Germany	19 (4.2%)	18 (4.0%)
Hungary	16 (3.5%)	17 (3.7%)
Portugal	15 (3.3%)	15 (3.3%)
Turkey	12 (2.6%)	12 (2.6%)
Greece	10 (2.2%)	10 (2.2%)
United Kingdom	9 (2.0%)	7 (1.5%)
France	8 (1.8%)	8 (1.8%)
Switzerland	8 (1.8%)	5 (1.1%)
Romania	7 (1.5%)	7 (1.5%)
Brazil	6 (1.3%)	6 (1.3%)
Poland	5 (1.1%)	7 (1.5%)
Austria	4 (0.9%)	5 (1.1%)
The Netherlands	5 (1.1%)	4 (0.9%)
Latvia	3 (0.7%)	3 (0.7%)
Denmark	2 (0.4%)	1 (0.2%)
Mexico	4 (0.9%)	3 (0.7%)
Russia	2 (0.4%)	2 (0.4%)
Belgium	2 (0.4%)	2 (0.4%)
Nigeria	2 (0.4%)	2 (0.4%)
Macedonia	3 (0.7%)	2 (0.4%)
Norway	1 (0.2%)	1 (0.2%)
Dominican Republic	1 (0.2%)	1 (0.2%)
Argentina	1 (0.2%)	1 (0.2%)
Costa Rica	1 (0.2%)	1 (0.2%)
Egypt	1 (0.2%)	1 (0.2%)
India	1 (0.2%)	1 (0.2%)
Indonesia	1 (0.2%)	1 (0.2%)
Northern Ireland	1 (0.2%)	1 (0.2%)
Palestine	1 (0.2%)	1 (0.2%)
Southern Rhodesia	1 (0.2%)	1 (0.2%)
Saudi Arabia	1 (0.2%)	1 (0.2%)
Iran	1 (0.2%)	1 (0.2%)
Bosnia and Herzegovina	0 (0.0%)	2 (0.4%)
Ecuador	0 (0.0%)	1 (0.2%)
Moldova	0 (0.0%)	1 (0.2%)
Montenegro	0 (0.0%)	1 (0.2%)
Congo	0 (0.0%)	1 (0.2%)
Syria	0 (0.0%)	1 (0.2%)
Taiwan	0 (0.0%)	1 (0.2%)
Venezuela	0 (0.0%)	1 (0.2%)
United States	0 (0.0%)	1 (0.2%)
Belarus	0 (0.0%)	1 (0.2%)
Total	454 (100.0%)	454 (100.0%)

### Sub-analysis based on experience and country

When comparing the answers of radiology residents and certified radiologists, few significant differences were observed. Specifically, certified radiologists (83%) reported to be better informed about the appropriate use of CM than residents (72%, *χ*^2^ = 19.0723, *p* = 0.000761), as well as more confident in primary management of CM-induced ADR (37% vs 26%, *χ*^2^ = 6.6257, *p* = 0.010052); further, 75% certified radiologists correctly declared that CM injection is not contraindicated during breastfeeding compared to 55% residents (*χ*^2^ = 32.5657, *p* < 0.00001). Last, a higher percentage of residents (55%) performed contrast enhanced-ultrasound at their institution than certified radiologists (31%, *χ*^2^ = 24.2865, *p* < 0.00001).

The top five represented countries (based on radiology training institutions) were Italy (35.5%), Slovenia (9.9%), Croatia (6.4%), Serbia (5.3%), and Spain (4.6%). We have reported just the responses with substantial differences among the top five represented countries in Table 3.

**Table 3:** The responses with substantial differences between the top five represented countries

Answer	Italy	Slovenia	Croatia	Serbia	Spain
6. What percentage of CT/MR are performed with CM at your Institution in a year?					
< 50%	21 (13.0%)	14 (31.1%)	7 (24.1%)	2 (8.3%)	8 (38.1%)
> 50%	140 (87.0%)	31 (68.9%)	22 (75.9%)	22 (91.7%)	13 (61.9%)
7. How many different CM (molecules) do you usually use for CT in your practical activity?					
0	0 (0.0%)	0 (0.0%)	2 (6.9%)	0 (0.0%)	0 (0.0%)
1	10 (6.2%)	13 (28.9%)	6 (20.7%)	3 (12.5%)	6 (28.6%)
2	42 (26.1%)	15 (33.3%)	14 (48.3%)	7 (29.2%)	12 (57.1%)
3	65 (40.4%)	12 (26.7%)	4 (13.8%)	2 (8.3%)	2 (9.5%)
> 4	44 (27.3%)	5 (11.1%)	3 (10.3%)	12 (50.0%)	1 (4.8%)
8. How many different CM (molecules) do you usually use for MR in your practical activity?					
0	2 (1.2%)	0 (0.0%)	3 (10.3%)	1 (4.2%)	0 (0.0%)
1	6 (3.7%)	8 (17.8%)	7 (24.1%)	2 (8.3%)	7 (33.3%)
2	46 (28.6%)	27 (60.0%)	15 (51.7%)	8 (33.3%)	10 (47.6%)
3	53 (32.9%)	8 (17.8%)	2 (6.9%)	2 (8.3%)	4 (19.0%)
> 4	54 (33.5%)	2 (4.4%)	2 (6.9%)	11 (45.8%)	0 (0.0%)
9. Are you well informed about the appropriate use of CM in diagnostic or interventional procedures?					
Yes	132 (82.0%)	34 (75.6%)	9 (31.0%)	19 (79.2%)	15 (71.4%)
No	29 (18.0%)	11 (24.4%)	20 (69.0%)	5 (20.8%)	6 (28.6%)
10. How many severe adverse reactions did you witness during your working practice in the past 12 months?					
None	96 (59.6%)	32 (71.1%)	19 (65.5%)	6 (25.0%)	6 (28.6%)
Less than 5	59 (36.6%)	13 (28.9%)	10 (34.5%)	18 (75.0%)	15 (71.4%)
6 to 20	6 (3.7%)	0 (0.0%)	0 (0.0%)	0 (0.0%)	0 (0.0%)
12. Are you familiar with the safety protocols for managing adverse reactions to CM established by your national or international Societies of Radiology, Urology or Anesthesia?					
Yes	100 (62.1%)	37 (82.2%)	21 (72.4%)	20 (83.3%)	7 (33.3%)
No	61 (17.8%)	8 (17.8%)	8 (27.6%)	4 (16.7%)	14 (66.7%)
15. In case of history of severe adverse reaction to CM, do you have to choose an alternative diagnostic procedure?					
Always	51 (31.7%)	27 (60.0%)	12 (41.4%)	13 (54.2%)	9 (42.9%)
I don’t know	5 (3.1%)	2 (4.4%)	3 (10.3%)	2 (8.3%)	0 (0.0%)
Never	4 (2.5%)	3 (6.7%)	0 (0.0%)	0 (0.0%)	1 (4.8%)
Only in certain cases	101 (62.7%)	13 (28.9%)	14 (48.3%)	9 (37.5%)	11 (52.4%)
18. Is intravenous CM injection contraindicated during pregnancy?					
I am not sure	30 (18.6%)	6 (13.3%)	12 (41.4%)	9 (37.5%)	3 (14.3%)
No	44 (27.3%)	20 (44.4%)	8 (27.6%)	8 (33.3%)	12 (57.1%)
Only in certain cases	30 (18.6%)	9 (20.0%)	2 (6.9%)	4 (16.7%)	3 (14.3%)
Yes	57 (35.4%)	10 (22.2%)	7 (24.1%)	3 (12.5%)	3 (14.3%)
25. In CT angiography examinations, which of the following CM injection strategy do you use?					
A predetermined fixed amount	28 (17.4%)	12 (26.7%)	9 (31.0%)	17 (70.8%)	7 (33.3%)
Based on iodine delivery rate (IDR)	56 (34.8%)	6 (13.3%)	2 (6.9%)	0 (0.0%)	5 (23.8%)
Based on patient’s lean body weight	52 (32.3%)	18 (40.0%)	14 (48.3%)	5 (20.8%)	7 (33.3%)
Based on scan time	25 (15.5%)	9 (20.0%)	4 (13.8%)	2 (8.3%)	2 (9.5%)
27. How is calculated the amount of CM to be injected in CT examinations?					
Simple calculator	49 (30.4%)	13 (28.9%)	6 (20.7%)	11 (45.8%)	7 (33.3%)
Pre-fixed tables	28 (17.4%)	14 (31.1%)	10 (34.5%)	5 (20.8%)	6 (28.6%)
Predetermined fixed amount	21 (13.0%)	11 (24.4%)	9 (31.0%)	5 (20.8%)	6 (28.6%)
Smartphone apps	63 (39.1%)	3 (6.7%)	2 (6.9%)	3 (12.5%)	2 (9.5%)
Approximated on patient’s weight	0 (0.0%)	1 (2.2%)	0 (0.0%)	0 (0.0%)	0 (0.0%)
I don’t know	0 (0.0%)	1 (2.2%)	0 (0.0%)	0 (0.0%)	0 (0.0%)
Calculator at CT	0 (0.0%)	2 (4.4%)	2 (6.9%)	0 (0.0%)	0 (0.0%)
28. Are you aware of the advantages of the use of CM in ultrasound (CEUS) in specific settings when compared to contrast-enhanced CT/MRI?					
I am not sure	24 (14.9%)	4 (8.9%)	6 (20.7%)	10 (41.7%)	0 (0.0%)
No	23 (14.3%)	4 (8.9%)	1 (3.4%)	5 (20.8%)	5 (23.8%)
Yes	114 (70.8%)	37 (82.2%)	22 (75.9%)	9 (37.5%)	16 (76.2%)
29. Do you perform contrast-enhanced ultrasound (CEUS) at your Institution?					
Yes	105 (65.2%)	36 (80.0%)	15 (51.7%)	1 (4.2%)	8 (38.1%)
No	56 (34.8%)	9 (20.0%)	14 (48.3%)	23 (95.8%)	13 (61.9%)

## Discussion

The most important finding emerging from this study was that nearly all participants reported that their training on primary management of ADR to CM during residency was poor to average. The majority of young radiologists reported that they did not receive dedicated training for CM use and the management of severe ADR during their residency.

It is important to note that differences in training programs and healthcare systems should be considered, even if discrepancies may be undetected by surveys with a relatively low number of participants from some countries. Nevertheless, since the survey comprises a substantial number of responses from young radiologists and trainees from different countries, it provides an intriguing snapshot of the current state of knowledge regarding CM, particularly in Europe.

A relevant aspect that emerges from this survey is that, while most young radiologists have claimed to be well-informed about the appropriate use of CM (indications to premedication and risk factors for CM-induced ADR), the vast majority of them did not feel confident in the primary management of ADR following the administration of CM. Additionally, slightly less than half (41%) of the participants were unfamiliar with the safety protocols for managing ADR to CM established by scientific societies [14]. A study by Lightfoot et al. surveying 253 radiologists found that 89% did not know the concentration of epinephrine present in their contrast reaction kit and what equipment would be required for appropriate epinephrine administration [15]. Notably, corticosteroid prophylaxis has an incomplete mitigating effect on the incidence of allergic-like CM reactions in high-risk patients and does not appear to affect reaction severity [16]. As a matter of fact, it is recommended to change the CM molecule in patients with documented previous CM-induced ADRs [17]; furthermore, in patients with a previous severe ACR to CM in whom a new examination is requested [17], some scientific societies suggest that it could be appropriate to have the presence of an anesthesiologist in the CT room [18], given that “…Patients who have had a prior allergic-like reaction….to contrast medium have an approximately fivefold increased risk of developing a future allergic-like reaction if exposed to the same class of contrast medium again…” [19]. Furthermore, the heterogeneity of answers concerning the need to change the diagnostic examination after a severe ADR to CM highlights the urge for education improvement of young radiologists on this topic as advocated by most participants. The importance of this point is corroborated by the non-negligible frequency of CM-induced severe ADRs, since, in the last year, about 50% of respondents faced these reactions, that in some cases represent life-threatening emergencies. In addition, the different responses received from residents and certified radiologists highlight that the latter feel more confident and skilled in the use of CM and the management of ADR. It can be justified by the longer experience of certified radiologists in daily practice and by the need to improve their knowledge after residency to face and solve day-to-day issues. As a matter of fact, there is an urge to improve education on CM during both the residency and post-residency period.

If we turn our attention to the data about the use of CM, we observed extreme heterogeneity in replies regarding the CM injection strategy in both CT angiography and parenchymal examinations, particularly in the former. Further proof of the different approaches adopted by young radiologists, several different ways are applied to calculate the amount of CM to be administered, including simple calculators, smartphone apps, pre-fixed tables, and predetermined fixed amounts. To date, it is well established that the iodine delivery rate (IDR) is the main determinant of vessel contrast enhancement. It is easily obtained through the formula IDR = ([iodine concentration in mg I/mL]/1000) × injection rate in mL/s [20, 21]. Conversely, CM injection strategy in CT parenchymal examinations should be based on the patient’s lean body weight. Indeed, parenchyma enhancement is based on blood and extracellular fluid volume [22]. However, fat tissues are poorly perfused, thus scarcely contributing to CM distribution. For this reason, the calculation of iodine dose based on lean body weight is considered more reliable than total body weight to reach the optimal parenchymal contrast enhancement [23]. However, the calculation of lean body weight is not as straightforward as that of total body weight; therefore, it may be impractical for daily implementation in clinical practice, especially in high-throughput centers. In such cases, the selection of total body weight represents an acceptable compromise. Another noteworthy point is the indication and/or contraindication for the use of CM. Regarding this topic, we have found a wide heterogeneity of answers concerning pregnancy and breastfeeding. While characteristics of the employed CM and appropriateness of the indication are essential in pregnancy and lactation, many participants were not aware on the statements of current guidelines on the topic. In accordance with the European Society of Urogenital Radiology guidelines [14], iodinated and gadolinium-based CM injections are not contraindicated during breastfeeding and can be done in exceptional circumstances when contrast enhanced CT or MRI are strongly indicated during pregnancy. Notably, gadolinium-based CM are contraindicated during both pregnancy and breastfeeding when the mother is affected by renal impairment.

Looking at the responses from the top five most represented countries, we noted a few relevant differences. Initially, while most participants from Italy, Slovenia, Serbia, and Spain claimed to be well-informed about the appropriate use of CM, two-thirds of the Croatian respondents were not. Additionally, only one-third of the Spanish were acquainted with protocols for managing ADRs established by scientific societies compared with 62–83% of participants from the other countries. Lastly, contrast-enhanced ultrasound appears to be more common used in Slovenia, Italy, and Croatia than in Spain and Serbia. No substantial differences were observed regarding the other questions. Specifically, our findings point to a potential deficiency in residency training programs across many countries in Europe concerning specific training on the applications of CM use, optimal injection strategy, and the management of CM-induced adverse events. Almost all participants expressed a desire to enhance their knowledge in these areas, regardless of their country of residency.

Clinical implications of inadequate knowledge among radiologists regarding the use of CM and the management of ADR can have profound consequences for patient safety and overall healthcare outcomes. First, inappropriate CM injection strategies may lead to inadequate image quality, potentially decreasing the diagnostic power of contrast-enhanced imaging examinations. Then, lack of awareness concerning risk factors, proper patient screening, and appropriate premedication strategies may contribute to an increased incidence and severity of ADRs. Poorly managed adverse reactions can impact patient outcomes. Furthermore, failure in recognizing and promptly addressing contrast-induced nephropathy, allergic reactions, or other adverse events may lead to long-term consequences for patients. Therefore, a comprehensive and up-to-date understanding of contrast media utilization and ADR management is crucial for radiologists to ensure safe and effective CM delivery and to mitigate potential risks to patient well-being. Addressing the knowledge gaps among radiologists concerning CM use and the management of ADR requires a multifaceted approach aimed at continuous education and quality improvements. Instituting regular and updated training programs on CM safety is of paramount importance. Incorporating educational initiatives into radiology residency programs, continuing medical education courses, and workshops can help ensure that radiologists stay abreast of the latest developments in CM safety protocols. Collaborations between radiology departments, urologists, and anesthesiologists could facilitate interdisciplinary training sessions, fostering a comprehensive understanding of CM pharmacology and potential drug reactions. Furthermore, the improvement and dissemination of standardized CM injection protocols and the implementation of guidelines within healthcare institutions may serve as valuable resources for radiologists at the point of care. Under this perspective, the combined efforts of scientific societies and industries are essential to emphasize a culture of continuous learning, open communication, and the dissemination of best practices to collectively contribute to enhance the knowledge and competency of radiologists in CM utilization, and ultimately promoting patient safety and improvement of the overall quality of contrast-enhanced imaging procedures.

A few limitations of this survey must be pointed out. First, this was not an all-inclusive survey, since we did not take into account a number of factors related to different national health service systems. Furthermore, several countries, particularly beyond European borders, were under/unrepresented. On the other hand, numerous replies were received from Italy and Slovenia where there was a wide distribution of the questionnaire. Last, some of the questions related to injection protocols might have been variably interpreted. Preparing questions for a survey is not an easy task, and the understating of the question might change based on language, culture, or background. We tried to create questions that would be easily understandable for a first-year resident. Nevertheless, this does not change the overall results of this survey and we believe that this data represents a comprehensive snapshot of residents and young radiologists concerning the use of CM.

In conclusion, we have reported the results of a large international survey about the knowledge and self-perception of radiology residents and young certified radiologists concerning the use of CM. Our results suggest that training about CM use and management of CM-induced ADRs should be implemented. Moreover, more attention by residency programs and scientific societies is required on this aspect of radiology training. Several options could be considered to improve the education, including dedicated sessions during radiology conferences, dedicated training periods during residency, routine didactic lectures, interactive group sessions, and hands-on workshops, as well as greater support by pharmaceutical companies. This is what young radiologists need and request to feel more confident in their daily practice.

## Data Availability

The datasets used and analyzed during the current study are available from the corresponding author on reasonable request.

## Abbreviations

ADR: Adverse drug reaction
CM: Contrast media
IDR: Iodine delivery rate
